# Miocardite Aguda como Apresentação Inicial da Mutação da Desmoplaquina – Ampliando o Diagnóstico Diferencial

**DOI:** 10.36660/abc.20230541

**Published:** 2024-04-17

**Authors:** Hideki Koike, Amr Idris, Justin Berger, Victor Y. Cheng, Jay Sengupta, João L. Cavalcante

**Affiliations:** 1 Cardiovascular Imaging Research Center Minneapolis Heart Institute Foundation Minneapolis MN EUA Cardiovascular Imaging Research Center and Core Lab at Minneapolis Heart Institute Foundation, Minneapolis, MN – EUA; 2 Allina Health Minneapolis Heart Institute Abbott Northwestern Hospital Minneapolis MN EUA Allina Health Minneapolis Heart Institute at Abbott Northwestern Hospital, Minneapolis, MN – EUA; 3 Valve Science Center Minneapolis Heart Institute Foundation Minneapolis MN EUA Valve Science Center, Minneapolis Heart Institute Foundation, Minneapolis, MN – EUA

**Keywords:** Miocárdio, Cardiomiopatia Dilatada, Gadolínio

## Introdução

A cardiomiopatia genética relacionada à mutação da desmoplaquina (DSP) pode iniciar sua manifestação clínica com sintomas de dor torácica, juntamente com elevação transitória da troponina, configurando a primeira manifestação como miocardite aguda, às vezes em padrão recidivante.^1^ Está claro que os critérios revisados da Força-Tarefa para cardiomiopatia genética deixarão de lado muitos casos, particularmente aqueles com dominância do lado esquerdo. Este relato de caso destaca a compreensão de que a cardiomiopatia genética, particularmente com mutação DSP, pode se apresentar com miocardite aguda e a compreensão de que a ressonância magnética cardiovascular (RMC) poderia aumentar a possibilidade de cardiomiopatia arritmogênica e prever taquiarritmias ventriculares apesar da FEVE preservada.

## Relato de Caso

Uma mulher de 20 anos, previamente saudável, deu entrada no pronto-socorro com dor torácica com duração de uma semana. Ela tinha histórico familiar de miocardite em sua irmã gêmea. Seus sinais vitais eram normais, exceto taquicardia, e não havia achados notáveis em seu exame físico.

**Figura 1:**
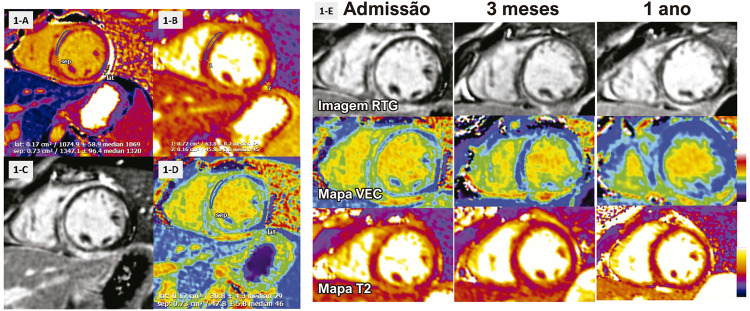
– Imagem miocárdica por RMC. Cada imagem mostra mapeamento T1 (1-A), mapeamento T2 (1-B), imagem de realce tardio com gadolínio (1-C) e mapa de volume extracelular (VEC) (1-D). A Figura 1-E mostra alterações seriadas na RMC ao longo de um ano, que demonstram a transição da necrose miocárdica + edema na apresentação inicial para a resolução do edema miocárdico com o passar do tempo e a transição para a fibrose miocárdica de substituição.

O eletrocardiograma mostrou taquicardia sinusal sem alterações isquêmicas (ritmo sinusal, frequência cardíaca 114 bpm). O peptídeo natriurético cerebral e o dímero D estavam dentro dos limites normais. Troponina I marcadamente elevada (TnI 9,7 ng/ml, normal < 0,034 ng/ml) motivou um ecocardiograma transtorácico, que mostrou tamanho do ventrículo esquerdo (VE) e função sistólica normais (FEVE=60%), e sem derrame pericárdico. A ressonância magnética cardiovascular (RMC) foi realizada no Siemens Aera 1,5T usando um protocolo clínico incluindo mapeamento T1 e T2 pré-contraste, imagem de cine, realce tardio com gadolínio (RTG) e mapeamento do volume extracelular (VEC). O estudo de RMC revelou tamanho normal do VE e função preservada (FEVE = 54%), sem anormalidades da contratilidade segmentar regional e tamanho e função normais do ventrículo direito (FEVD = 61%). A elevação pré-contraste do T1 miocárdico nativo (Figura 1-A, T1>1050 mseg) sugeriu edema e/ou aumento do espaço intersticial (fibrose prévia), enquanto a elevação do T2 miocárdico (Figura 1-B, T2>55 mseg) foi consistente com edema envolvendo predominantemente o septo médio. A imagem do RTG mostrou um padrão não isquêmico em forma de anel envolvendo o subepicárdio e o miocárdio médio nos segmentos médio e apical anterior, septo e inferior (Figura 1-C). O volume extracelular miocárdico estava notavelmente elevado (46%, Figura 1-D). Não houve derrame pericárdico nem realce pericárdico. Os achados gerais foram consistentes com lesão miocárdica aguda não isquêmica, conforme descrito nos critérios atualizados de miocardite de Lake-Louise.^2^ Os exames do painel viral, incluindo COVID-19, foram negativos.

**Figura 2:**
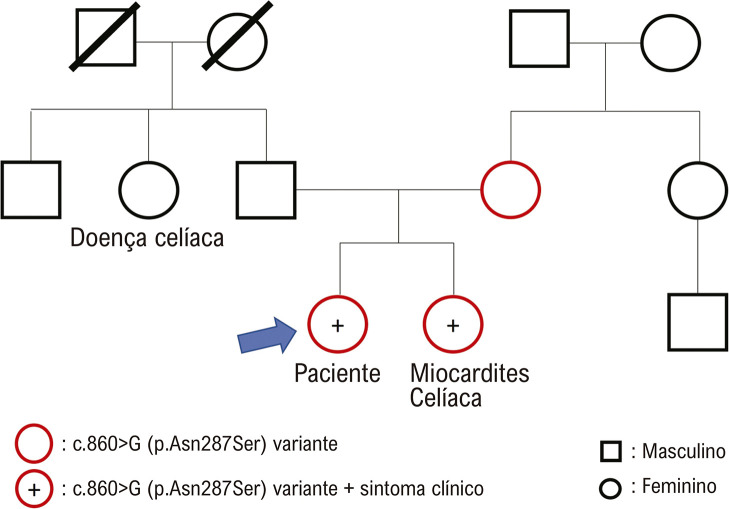
– Linhagem Familiar da Mutação da Desmoplaquina. A caixa preta e o círculo mostram os membros da família sem mutação. Vermelho indica indivíduos com genes mutados e vermelho mais (+) indica indivíduos com sintomas clínicos. Embora o teste do painel genético tenha revelado a variante de significado incerto (VUS) no gene DSP (c.860A>G [p.Asn287Ser]), a mesma mutação foi encontrada em sua mãe e irmã.

Contrações ventriculares prematuras (CVPs) e taquicardia ventricular não sustentada (TVNS) foram observadas em um monitor cardíaco, para o qual um betabloqueador foi iniciado e titulado. A ressonância magnética de acompanhamento, 3 e 12 meses após a apresentação inicial, revelou resolução do edema miocárdico, mas persistência da fibrose miocárdica (Figura 1-E). Devido ao histórico familiar de miocardite, foi obtido aconselhamento genético. Sua irmã também apresentou dor torácica e elevação de troponina I (4,16 ng/ml) e foi diagnosticada com miocardite aguda como possível fenótipo dessa variante. Embora a função do VE fosse normal (FEVE 56%, VCE 26%), o RTG foi identificado no septo médio-inferior e na parede inferior. Um painel de testes genéticos identificou uma mutação missense que leva a uma variante de significado desconhecido no gene DSP, que está associada à cardiomiopatia dilatada e à cardiomiopatia arritmogênica do ventrículo direito. A presença da mesma variante DSP (c.860A> G [p.Asn287Ser]) na irmã do paciente, que já havia apresentado miocardite, aumentou substancialmente nossa suspeita de que a variante era bastante patológica (Figura 2).

O paciente recebeu alta hospitalar e acompanhamento ambulatorial no ambulatório de eletrofisiologia com posterior monitorização arrítmica por meio de eletrocardiograma Holter estendido. Ela descreveu melhora significativa em seus sintomas após a alta. Durante o seguimento (3 anos), o Holter estendido revelou um episódio de TVNS (6 batimentos) e carga de 1,1% de CVPs sob betabloqueador (carvedilol 6,25 mg duas vezes ao dia). Porém, recentemente, voltou a apresentar pressão torácica, pré-síncope e elevação de troponina, porém com ritmo sinusal no eletrocardiograma. A implantação de um cardioversor desfibrilador implantável (CDI) foi realizada para prevenção secundária, devido à TVNS, aos sintomas pré-sincopais e à alta probabilidade de TV recorrente.

## Discussão

A maioria dos casos de miocardite aguda é causada por um gatilho viral ou por uma etiologia autoimune. No entanto, uma predisposição genética também foi relatada.^1^ Nesses pacientes, uma história familiar positiva de miocardite ou morte cardíaca súbita pode levar a testes genéticos. Um estudo recente demonstrou que a distribuição do RTG na RMC identificou os pacientes de maior risco entre aqueles com FEVE preservada.^3^

Este caso destaca a manifestação inicial da cardiomiopatia DSP como uma causa incomum de miocardite aguda. Embora o tratamento da miocardite secundária à cardiomiopatia DSP não esteja totalmente acordado, os sintomas de dor torácica, juntamente com a elevação da troponina e o extenso padrão de fibrose não isquêmica na RMC, devem levar à consideração desta entidade.^4,5^ Particularmente, o padrão de RTG não isquêmico subepicárdico e de parede média em forma de anel está associado à possibilidade de cardiomiopatia arritmogênica e taquiarritmias ventriculares, mesmo com FEVE preservada.^3,6^ Esses achados devem levar à consideração da implantação de um desfibrilador intracardíaco ou cardioversor desfibrilador implantável subcutâneo para terapia de prevenção primária. Finalmente, neste caso, o acompanhamento e monitoramento em longo prazo revelaram a necessidade de implantação de CDI para prevenção secundária. O acompanhamento ambulatorial longitudinal e a repetição de imagens de RMC podem fornecer informações valiosas sobre a progressão da doença e orientar futuras intervenções terapêuticas.

### Limitações

A variante genética identificada no gene DSP é atualmente classificada como variante de significado incerto (VUS), indicando evidências insuficientes para o papel da mutação. Na verdade, esta mutação foi detectada na paciente, na sua irmã e na sua mãe. Porém, manifestações clínicas foram observadas apenas na paciente e em sua irmã. Um número insuficiente de portadores e fenótipos dentro da família impede uma ligação conclusiva entre este VUS e o fenótipo clínico observado. Dada esta limitação, é necessária uma investigação mais ampla da co-segregação na família no futuro para fornecer evidências mais fortes que apoiem a patogenicidade da variante.

## Conclusão

Neste caso, o protocolo de imagem de RMC utilizando mapas miocárdicos e RTG foi crucial não só para o diagnóstico de miocardite, mas também para o acompanhamento. Além disso, a mesma mutação genética foi detectada na mãe e na irmã, enfatizando a importância da informação da história familiar em pacientes que apresentam miocardite, dada a potencial base genética, particularmente para a mutação DSP.

## References

[1] McNally EM, Selgrade DF (2022). Genetic Testing for Myocarditis. JACC Heart Fail.

[2] Ferreira VM, Schulz-Menger J, Holmvang G, Kramer CM, Carbone I, Sechtem U (2018). Cardiovascular Magnetic Resonance in Nonischemic Myocardial Inflammation: Expert Recommendations. J Am Coll Cardiol.

[3] Chen W, Qian W, Zhang X, Li D, Qian Z, Xu H (2021). Ring-Like Late Gadolinium Enhancement for Predicting Ventricular Tachyarrhythmias in Non-Ischaemic Dilated Cardiomyopathy. Eur Heart J Cardiovasc Imaging.

[4] Smith ED, Lakdawala NK, Papoutsidakis N, Aubert G, Mazzanti A, McCanta AC (2020). Desmoplakin Cardiomyopathy, a Fibrotic and Inflammatory Form of Cardiomyopathy Distinct from Typical Dilated or Arrhythmogenic Right Ventricular Cardiomyopathy. Circulation.

[5] Reichl K, Kreykes SE, Martin CM, Shenoy C (2018). Desmoplakin Variant-Associated Arrhythmogenic Cardiomyopathy Presenting as Acute Myocarditis. Circ Genom Precis Med.

[6] Ammirati E, Raimondi F, Piriou N, Infirri LS, Mohiddin SA, Mazzanti A (2022). Acute Myocarditis Associated with Desmosomal Gene Variants. JACC Heart Fail.

