# Supplementary material for: Miocardite Aguda como Apresentação Inicial da Mutação da Desmoplaquina – Ampliando o Diagnóstico Diferencial
Source: Arq Bras Cardiol. 2024 Apr 17;121(4):e20230541. [Article in Portuguese] doi: 10.36660/abc.20230541 (PMC11098571; doi:10.36660/abc.20230541)
Supplement: Translation

# Translation: Acute Myocarditis as the Initial Presentation of Desmoplakin Mutation – Broadening the Differential Diagnosis

**DOI:** 10.36660/abc.20230541i

**Authors:** Hideki Koike, Amr Idris, Justin Berger, Victor Y. Cheng, Jay Sengupta, João L. Cavalcante

**Affiliations:** 1 USA Cardiovascular Imaging Research Center and Core Lab at Minneapolis Heart Institute Foundation, Minneapolis, MN – USA; 2 USA Allina Health Minneapolis Heart Institute at Abbott Northwestern Hospital, Minneapolis, MN – USA; 3 USA Valve Science Center, Minneapolis Heart Institute Foundation, Minneapolis, MN – USA

**Keywords:** Myocardium, Dilated Cardiomyopathy, Gadolinium

## Introduction

Genetic cardiomyopathy related to desmoplakin (DSP) mutation can start its clinical manifestation with chest pain symptoms, along with transient troponin elevation configuring first manifest as acute myocarditis, sometimes in a relapsing pattern.^1^ It is clear that the revised Task Force criteria for genetic cardiomyopathy will miss many cases, particularly those with left-sided dominance. This case reports highlights to understand that genetic cardiomyopathy, particularly with DSP mutation, can present with acute myocarditis and to understand that cardiovascular magnetic resonance (CMR) imaging could raise the possibility of arrhythmogenic cardiomyopathy and predict ventricular tachyarrhythmias despite preserved LVEF.

## Case Report

A previously healthy 20-year-old female presented to the emergency room with chest pain of one-week duration. She had a family history of myocarditis in her twin sister. Her vital signs were normal except for tachycardia, and there were no remarkable findings on her physical exam.

The electrocardiogram showed sinus tachycardia with no ischemic changes (Sinus rhythm, heart rate 114 bpm). Brain natriuretic peptide and D-dimer were within normal limits. Markedly elevated troponin I (TnI 9.7 ng/ml, normal < 0.034 ng/ml) prompted a transthoracic echocardiogram, which showed normal left ventricular (LV) size and systolic function (LVEF=60%), and no pericardial effusion. Cardiovascular magnetic resonance (CMR) was performed in the 1.5T Siemens Aera using a clinical protocol including pre-contrast T1 and T2 mapping, cine imaging, late gadolinium enhancement (LGE), and extracellular volume (ECV) mapping. CMR study revealed normal LV size and preserved function (LVEF=54%) without regional wall motion abnormalities and normal right ventricular size and function (RVEF=61%). Pre-contrast elevation of myocardial native T1 (Figure 1-A, T1>1050 msec) suggested edema and/or increase of interstitial space (previous fibrosis), while elevation of myocardial T2 (Figure 1-B, T2>55 msec) was consistent with edema predominantly involving the mid septum. LGE imaging showed a ring-like non-ischemic pattern involving the subepicardium and mid-myocardium in the mid and apical anterior, septum, and inferior segments (Figure 1-C). Myocardial extracellular volume was remarkably high (46%, Figure 1-D). There was no pericardial effusion and no pericardial enhancement. Overall findings were consistent with acute non-ischemic myocardial injury as described in the updated Lake-Louise myocarditis criteria.^2^ Viral panel examinations, including COVID-19, were negative.

**Figure 1:** – CMR myocardial imaging. Each image shows T1 mapping (1-A), T2 mapping (1-B), late gadolinium enhancement image (1-C), and extracellular volume (ECV) map (1-D). Figure1-E shows serial CMR changes over the course of one year, which demonstrate the transition from myocardial necrosis+edema at the initial presentation to overtime resolution of myocardial edema and transition to replacement myocardial fibrosis.

Premature ventricular contractions (PVCs) and non-sustained ventricular tachycardia (NSVT) were seen on a cardiac monitor, for which a beta-blocker was initiated and uptitrated. Follow-up CMR imaging, at 3 and 12 months after initial presentation, revealed resolution of myocardial edema but persistency of myocardial fibrosis (Figure 1-E). Because of her family history of myocarditis, genetic counseling was obtained. Her sister also presented with chest pain and Troponin I elevation (4.16 ng/ml) and was diagnosed with acute myocarditis as a possible phenotype of this variant. Although her LV function was normal (LVEF 56%, ECV 26%), LGE was identified in the mid-inferior septum and inferior wall. A genetic testing panel identified a missense mutation leading to a variant of unknown significance in the DSP gene, which is associated with dilated cardiomyopathy and arrhythmogenic right ventricular cardiomyopathy. The presence of the same DSP variant (c.860A>G [p.Asn287Ser]) in the patient’s sister, who had already experienced myocarditis, substantially increased our suspicion that the variant was rather pathologic (Figure 2).

**Figure 2:** – Family-Pedigree of Desmoplakin Mutation. Black box and circle show family members without mutation. Red indicates individuals with mutated genes, and red plus (+) indicates individuals with clinical symptoms. While the genetic panel test revealed the variant of uncertain significance (VUS) in the DSP gene (c.860A>G [p.Asn287Ser]), the same mutation was found in her mother and sister.

The patient was discharged home and received outpatient follow-up care in the electrophysiology clinic with further arrhythmic monitoring using with extended Holter electrocardiogram. She described significant improvement in her symptoms after her discharge. During the follow-up (3 years), extended Holter revealed one episode of NSVT (6 beats) and 1.1% burden of PVCs under beta-blocker (carvedilol 6.25mg two times a daily). However, recently, she presented again with chest pressure, presyncope, and troponin elevation, albeit with sinus rhythm on the electrocardiogram. Implantation of an implantable cardioverter defibrillator (ICD) was performed for secondary prevention, given NSVT, presyncopal symptoms, and the high likelihood of recurrent VT.

## Discussion

Most cases of acute myocarditis are caused by a viral trigger or an autoimmune etiology. However, a genetic predisposition has also been reported.^1^ In those patients, a positive family history of myocarditis or sudden cardiac death can lead to genetic testing. A recent study demonstrated that the distribution of LGE on CMR identified the higher-risk patients within those with preserved LVEF.^3^

This case highlights the initial manifestation of DSP cardiomyopathy as an unusual cause of acute myocarditis. While the management of myocarditis secondary to DSP cardiomyopathy is not fully agreed upon, chest pain symptoms, along with troponin elevation and extensive non-ischemic fibrosis pattern on CMR, should prompt the consideration for this entity.^4,5^ Particularly, the ring-like subepicardial and midwall non-ischemic LGE pattern is associated with the possibility of arrhythmogenic cardiomyopathy and ventricular tachyarrhythmias, even with preserved LVEF.^3,6^ These findings should prompt the consideration of implanting an intracardiac defibrillator or subcutaneous implantable cardioverter defibrillator for primary prevention therapy. Finally, in this case, long-term follow-up and monitoring revealed the necessity for ICD implantation for secondary prevention. Longitudinal outpatient follow-up care and repeated CMR imaging can provide valuable insights into disease progression and guide further therapeutic interventions.

### Limitations

The genetic variant identified in the DSP gene is currently classified as a variant of uncertain significance (VUS), indicating insufficient evidence for the role of the mutation. Indeed, this mutation was detected in the patient, her sister, and her mother. However, clinical manifestations were observed only in the patient and her sister. An insufficient number of carriers and phenotypes within the family preclude a conclusive link between this VUS and the observed clinical phenotype. Given this limitation, a broader investigation of co-segregation in the family is needed in the future to provide stronger evidence supporting the pathogenicity of the variant.

## Conclusion

In this case, the CMR imaging protocol using myocardial maps and LGE was crucial not only for the diagnosis of myocarditis but also for the follow-up. Furthermore, the same genetic mutation was detected in her mother and sister, emphasizing the importance of family history information in patients presenting with myocarditis, given the potential genetic basis, particularly for DSP mutation.
